# Experimental Study on Bottom-Up Detection of Underwater Targets Based on Polarization Imaging

**DOI:** 10.3390/s22082827

**Published:** 2022-04-07

**Authors:** Tianfeng Pan, Xianqiang He, Xuan Zhang, Jia Liu, Yan Bai, Fang Gong, Teng Li

**Affiliations:** 1 Ocean College, Zhejiang University, Zhoushan 316021, China; pantianfeng@zju.edu.cn; 2 State Key Laboratory of Satellite Ocean Environment Dynamics, Second Institute of Oceanography, Ministry of Natural Resources, Hangzhou 310012, China; zhangxuan@sio.org.cn (X.Z.); liujia1@opt.ac.cn (J.L.); baiyan@sio.org.cn (Y.B.); gongfang@sio.org.cn (F.G.); liteng@sio.org.cn (T.L.); 3 Southern Marine Science and Engineering Guangdong Laboratory (Guangzhou), Guangzhou 511458, China; 4 Key Laboratory of Spectral Imaging Technology of CAS, Xi’an Institute of Optics and Precision Mechanics of the Chinese Academy of Sciences, Xi’an 710119, China

**Keywords:** underwater polarization imaging, targets, polarization parameters, bottom-up observation, angle of polarization (AOP)

## Abstract

Previous studies on the polarization imaging of underwater targets mainly focused on top-down detection; however, the capacities of bottom-up detection were poorly known. Based on in situ experiments, the capability of bottom-up detection of underwater targets using polarization imaging was investigated. First, to realize the objective of bottom-up polarization imaging, a SALSA polarization camera was integrated into our Underwater Polarization Imaging System (UPIS), which was integrated with an attitude sensor. At Qiandao Lake, where the water is relatively clear, experiments were conducted to examine the capacity of the UPIS to detect objects from the bottom up. Simultaneously, entropy, clarity, and contrast were adopted to compare the imaging performance with different radiation parameters. The results show that among all the used imaging parameters, the angle of polarization is the optimal parameter for bottom-up detection of underwater targets based on polarization imaging, which may result from the different diffused reflectance of the target surface to the linear polarization components of the Stokes vector.

## 1. Introduction

Because of its high imaging quality, large amount of information, and intuitive display, underwater optical imaging technology is widely used in intelligent assisted drowning-detection systems [1], marine aquaculture [2], underwater archaeology [3] and other fields [4]. Currently, methods of passive optical imaging of underwater targets in natural light in the field can be divided into intensity-only imaging and polarization imaging. Because radiance transmission is easily affected by particles in the water, intensity-only imaging results are of poor quality [4,5]. Compared with intensity-only imaging, polarization imaging is less affected by the scattering of particles in water. Tyo et al. [6] quantitatively compared polarization difference imaging (PDI) and intensity-only (or polarization-sum) imaging and found that compared with intensity-only imaging, PDI could suppress partially polarized background variations selectively and yield a factor of 2–3 increase in the distance at which certain target features could be detected. In practice, there is a significant difference in the state of polarization between the light reflected by targets and the light scattered by the medium, which gives rise to the higher imaging quality of polarization imaging compared to that of intensity-only imaging [7]. Amer et al. [8] used a polarimetric imaging optical system to reduce the effect of diffusion on the underwater image acquisition and reduced runtime by a factor of about 50 for a 4K image when compared to the conventional dark channel prior methods. Van der Laan et al. [9] quantified both linearly and circularly polarized active illumination and showed that circular polarization persisted better than linear for radiation fog in the short-wave infrared and large particle sizes of Sahara dust around the 4 μm wavelength. As a consequence, owing to its excellent performance, polarization imaging technology has been applied in many fields (e.g., active optical polarization imaging [10], passive infrared polarization imaging [11], and microwave radar [12]).

Considerable research has been conducted on how to maximize the difference between the polarized light containing the target information and its background light field so that the target can be identified. Schechner et al. [13] proposed a passive underwater polarization imaging model in which the scene was reconstructed by using the difference of polarization characteristics between background and target. Moreover, based on the analysis results of the polarization statistics of the scene, Yemelyanov et al. [14] proposed adaptive algorithms through which the optimal polarization direction of the underwater imaging was predicted and the image contrast was significantly improved. Underwater target imaging is affected by the type of target, and the polarization states of light reflected on the surface of different targets are also different. Watkins et al. [15] pointed out that artificial objects often contain more polarization information than natural objects. Cooper et al. [16] conducted polarization detection experiments at sea with ships as targets and found that the polarization characteristics of different targets in different bands were significantly different. They also found that the polarization degree of imaging increases with the increase in the observation angle but decreases with the increase in target roughness. In addition, the inherent optical properties of the medium also play a significant factor affecting underwater polarization imaging. Lewis et al. [17] used cross-polarization to detect targets in turbid water and calculated the corresponding contrast based on the polarization information of targets, which was more distinguishable than intensity-only imaging. In general, numerous studies have been conducted on polarization detection and polarization image processing, but they are mainly concentrated in the laboratory environment and the means by which polarization images are obtained is also basically in the form of top-down detection. However, to date, there are few studies on the real underwater environment, and there is a lack of research on underwater polarization imaging for bottom-up target detection.

For the purpose of exploring the ability of underwater polarization imaging from the bottom up, we developed an Underwater Polarization Imaging System (UPIS) in this research. Experiments were conducted at Qiandao Lake with natural lighting, where the water was relatively clear. Using this UPIS, we examined the performance of bottom-up detection of underwater artificial targets based on intensity-only imaging and imaging with different polarization parameters. Meanwhile, the radiation transmission mechanism was analyzed based on the experimental results.

## 2. Data and Methods

### 2.1. UPIS

**Figure 1:**
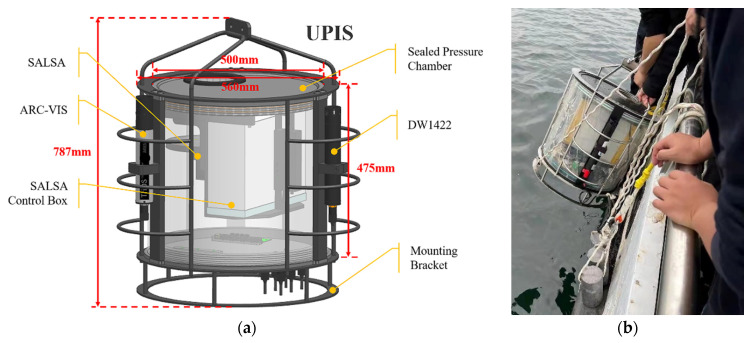
UPIS and in situ experiment. (**a**) Dimensions and components of the UPIS; (**b**) UPIS in situ experiment.

To conduct the underwater polarization imaging, a UPIS was designed and assembled, as shown in Figure 1. The weight of UPIS in the air is about 45 kg. At the same time, due to the influence of water buoyancy, a counterweight of 28 kg needs to be added when lowering on site. The SALSA polarization camera, ARC-VIS hyperspectral radiometer, DW1422 thermometer, and attitude sensor were integrated into the UPIS. In the field experiment, the UPIS was placed at the designated water depth. All sensors were connected to the deck unit by communication cables. SALSA data (*S*_0_, *S*_1_, *S*_2_, *S*_3_, DOP, DOLP, DOCP, AOP, and *ε*), ARC-VIS data (320–950 nm radiance spectrum), DW1422 data (temperature and depth), and attitude sensor parameters (X, Y, and inclination) were displayed in real time in computer software. The entire UPIS can meet the requirement of 50 m water depth. The SALSA camera was encapsulated in a sealed cabin, and a nonbirefringent glass (BK7, UVFS, K9, etc.) was embedded in the corresponding position its light reception part as the compression window, which does not change the polarization state of the incident light. Since the FOV (the field of view) hardly changed the texture characteristics in the targets imaging, we did not consider the FOV difference caused by the different refractive index between water and air here.

**Figure 2:**
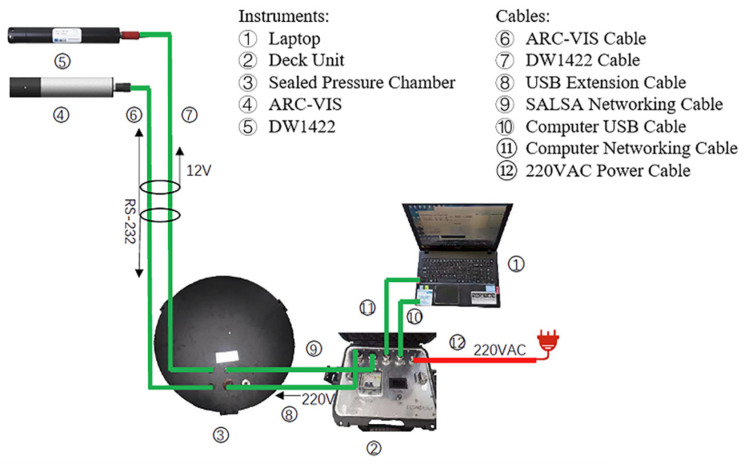
UPIS cable connections.

Details of the communication cable connections are shown in Figure 2. The instruments used in the experiment were the laptop equipped with SALSA version 2.3.6, the SALSA camera, a hyperspectral radiometer, a thermometer, and other devices. The major camera parameters are listed in Table 1. In the experiment, considering that blue–green light has the higher penetrate capacity in the clear and productive waters [18], a 550 nm sampling band was adopted to image underwater targets.

### 2.2. Polarization Measurement Using the UPIS

To describe the polarization state of radiation in a given direction, we adopt the Stokes vector convention as follows [19]:

(1) $$S=\left[\begin{array}{c} \begin{array}{c} S_{0}\\ S_{1} \end{array}\\ \begin{array}{c} S_{2}\\ S_{3} \end{array} \end{array}\right]=\left[\begin{array}{c} \begin{array}{c} \langle {\left|E_{x}\right|}^{2}\rangle +\langle {\left|E_{y}\right|}^{2}\rangle \\ \langle {\left|E_{x}\right|}^{2}\rangle -\langle {\left|E_{y}\right|}^{2}\rangle \end{array}\\ \begin{array}{c} \langle 2E_{x}E_{y}\cos\delta \rangle \\ \langle 2E_{x}E_{y}\sin\delta \rangle \end{array} \end{array}\right]$$

where *S*_0_ is the total light intensity detected by the UPIS, *S*_1_ is the linearly polarized component in the meridian plane or perpendicular to the meridian plane, *S*_2_ is the linearly polarized component in the direction 45° or 135° to the meridian plane, *S*_3_ is the light intensity difference between the left- and right-handed polarized light, *E*_x_ and *E*_y_ are the components of the electric vector along the *X* and *Y* directions, respectively, in the selected coordinate system, *δ* is the phase difference between *E*_x_ and *E*_y_, and the notation 〈 〉 represents the time average For light in water or on the water surface, *S*_3_ is negligible.

The SALSA allows switching polarization state between four linear polarization states (−45°, 0°, 45° and 90°) [20]. However, the polarization states are not perfectly −45°, 0°, 45° and 90°, so the Stokes parameters (*S*_0_, *S*_1_, *S*_2_, *S*_3_) are calculated for each pixel of the imaged scene by multiplying the calibration matrix [21]:

(2) $$\vec{S}=C\cdot \vec{I},$$

(3) $$\vec{S}=\left[\begin{array}{cccc} 0.1935 & 0.3555 & 0.3249 & 0.1346\\ 1.2827 & -1.1338 & -0.8719 & 0.7037\\ 0.8640 & -1.0428 & -1.6195 & 1.7759\\ -0.2958 & -0.4399 & 0.5381 & 0.2014 \end{array}\right]\times \left[\begin{array}{c} I_{Frame0}\\ I_{Frame1}\\ I_{Frame2}\\ I_{Frame3} \end{array}\right],$$

where the condition number of C is 6.7. With the 4 polarization images acquired in real time, it is able to calculate the linear Stokes parameters and other polarization parameters.

In the UPIS, the SALSA camera is used for the imaging measurement of the Stokes vector, and the images of degree of polarization (DOP), degree of linear polarization (DOLP), degree of circular polarization (DOCP), angle of polarization (AOP), and ellipticity angle (ε) are further calculated. These parameters are defined as follows [21,22,23]:

1. DOP represents the ratio of polarized part (linear polarization and circular polarization) to the total intensity of received light. It is calculated as follows:

(4) $$\mathrm{DOP}=\frac{\sqrt{S_{1}^{2}+S_{2}^{2}+S_{3}^{2}}}{S_{0}}\times 100\%,$$

2. DOLP refers to the ratio of the linearly polarized part to the total intensity of received light. It is calculated as follows:

(5) $$\mathrm{DOLP}=\frac{\sqrt{S_{1}^{2}+S_{2}^{2}}}{S_{0}}\times 100\%,$$

3. DOCP refers to the ratio of the circularly polarized part to the total intensity of received light. It is calculated as follows:

(6) $$\mathrm{DOCP}=\left|\frac{S_{3}}{S_{0}}\right|\times 100\%,$$

4. AOP denotes the azimuthal angle relative to elliptical polarization when the light is partially or totally linearly polarized. It is calculated (in units of degrees) as follows:

(7) $$\mathrm{AOP}=\frac{1}{2}\arctan\frac{S_{2}}{S_{1}},$$

5. *ε* denotes the elliptic angle relative to elliptical polarization when the light is partially or totally linearly polarized. It is calculated (in units of degrees) as follows:

(8) $$\varepsilon =\frac{1}{2}\arcsin\frac{S_{3}}{S_{0}},$$

The values of *S*, DOP, DOLP, DOCP, AOP, and *ε* obtained by the SALSA camera can be adopted to calculate the entropy, clarity, and contrast of targets, thereby realizing target recognition.

### 2.3. Evaluation Index of Image Quality

After obtaining data with the UPIS, we can evaluate the imaging quality of the collected polarization images visually. Although the visual evaluation is simple and intuitive, it depends too much on the subjective feeling of observers. Different people have different opinions on the same image. For example, while imaging, even if the contrast values calculated of the two images are the same, people may think that the contrast of the two images is different when judging subjectively. Therefore, only subjective evaluation can be used as a reference for real-time observation of imaging quality in the experiment. When analyzing the data collected by the UPIS, it is essential to determine the objective evaluation index to judge the imaging quality of target detection. We adopted the entropy, clarity, and contrast of each group of images as the criteria for evaluating image quality. These indexes are defined as follows:

1. Entropy. The entropy reflects the amount of information contained in the corresponding image. When the image has only one color, that is, the image contains only one gray value, the entropy of this image is 0. When the gray value of each pixel is different, the entropy is maximum. The entropy equation for a gray image is [24]:

(9) $$I=-\sum_{i=0}^{N}P\left(i\right)\log_{2}P\left(i\right),$$

where *P*(*i*) is the probability of a pixel value *i* appearing in the image.

2. Clarity. We obtain the gradient matrix *G* (*x*, *y*) by convoluting the Laplace operator and the gray value of each pixel in the image and taking the sum of squares of *G* (*x*, *y*) as the clarity evaluation function. Therefore, clarity can be written in terms of the gradient matrix *G* (*x*, *y*) as [25]:

(10) $$F=\sum_{x}\sum_{y}G^{2}\left(x,y\right),$$

where G(x,y)=f(x,y)⊗L and $L=\left[\begin{array}{ccc} 0 & 1 & 0\\ 1 & -4 & 1\\ 0 & 1 & 0 \end{array}\right]$.

3. Contrast. The evaluation index of different brightness levels of the image is contrast. The higher the brightness level, the higher the contrast. Contrast reflects the level at which the details in the image can be distinguished by the naked eye. The formula is as follows [26]:

(11) $$C=\sum_{\delta }\delta {\left(i,\;j\right)}^{2}P_{\delta }\left(i,j\right),$$

where δ(i,j)=|i−j| refers to the gray difference between adjacent pixels and $P_{\delta }\left(i,j\right)$ refers to the distribution probability of δ(i,j).

## 3. Underwater Target Detection Using the UPIS

**Figure 3:**
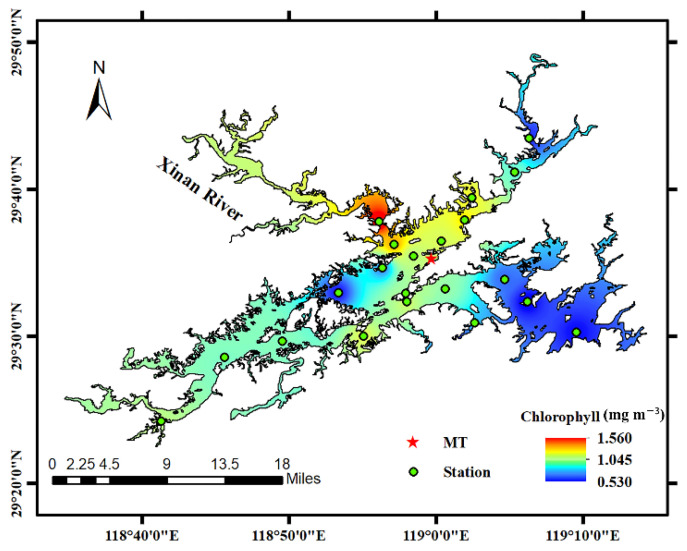
Distribution of sampling sites in Qiandao Lake and the corresponding interpolation of chlorophyll concentration. The green solid circles with black edges represent the sampling sites. The red solid five-pointed star corresponds to the experimental location of polarization target detection (where *C*_chl_ = 1.02 mg m^−3^).

From 9:00 to 13:00 on 29 October 2021, we conducted the target detection experiment using the UPIS at Qiandao Lake. The Qiandao Lake is located at Chun’an County, which is at the west of Zhejiang Province, China (29°22′–9°50′ N, 118°34′–119°15′ E, Figure 3). It is one of the largest reservoirs in China with a surface water area of 573 km^2^ and a water capacity of 178.4 × 108 m^3^. The east–west length of Qiandao Lake is about 150 km and the widest is 10 km. There are 34 inflow tributaries around the lake. The largest one (Xin’an River) is from the northwest, carrying 51.4% of the total inflow to the lake from all sources. Qiandao Lake is a deep water lake, and in particular the mean water depth is 34 m and the maximum depth is 100 m [27]. The water quality of Qiandao Lake is clear and transparent for ~5 m [28].

Light field conditions were generally excellent during the experiment, with a small amount of cloud cover. Figure 3 shows the distribution of sites used in this experiment. Considering the convenience and safety of the experimental process, the UPIS experiment was conducted at MT site. Meanwhile, we measured chlorophyll concentration at 21 other sites to obtain the chlorophyll concentration distribution of Qiandao Lake. The sampling results showed that the chlorophyll concentration of the MT site was 1.02 mg m^−3^ and water depth was 8 m.

Two groups of experiments were conducted using the UPIS to detect underwater targets from bottom up under natural lighting: Experiment 1, in which the same target was used at different depths, and Experiment 2, in which different targets were used at the same depth.

These two groups of experiments were used to examine the ability of the UPIS to image targets from bottom up under different experimental conditions. Changing the water depth of the UPIS and the target can enable us to test the strength of the UPIS to detect the target from bottom up under different natural light intensities. Changing the types of artificial targets reflects the detection capability of the UPIS for targets with different surface diffuse reflectance values.

At the beginning of the experiment, the UPIS was assembled on the deck to check the waterproofness of the sealing cabin equipped with the SALSA camera. Then, the UPIS was lowered to the pre-designed water depth and fixed vertically. The lens of the SALSA camera was perpendicular to the water surface, and the field of vision was kept far away from the shadow of the ship as much as possible. Moreover, the target was positioned to the pre-designed depth. By moving the target, the image of interest gradually becomes sharper in the software.

**Figure 4:**
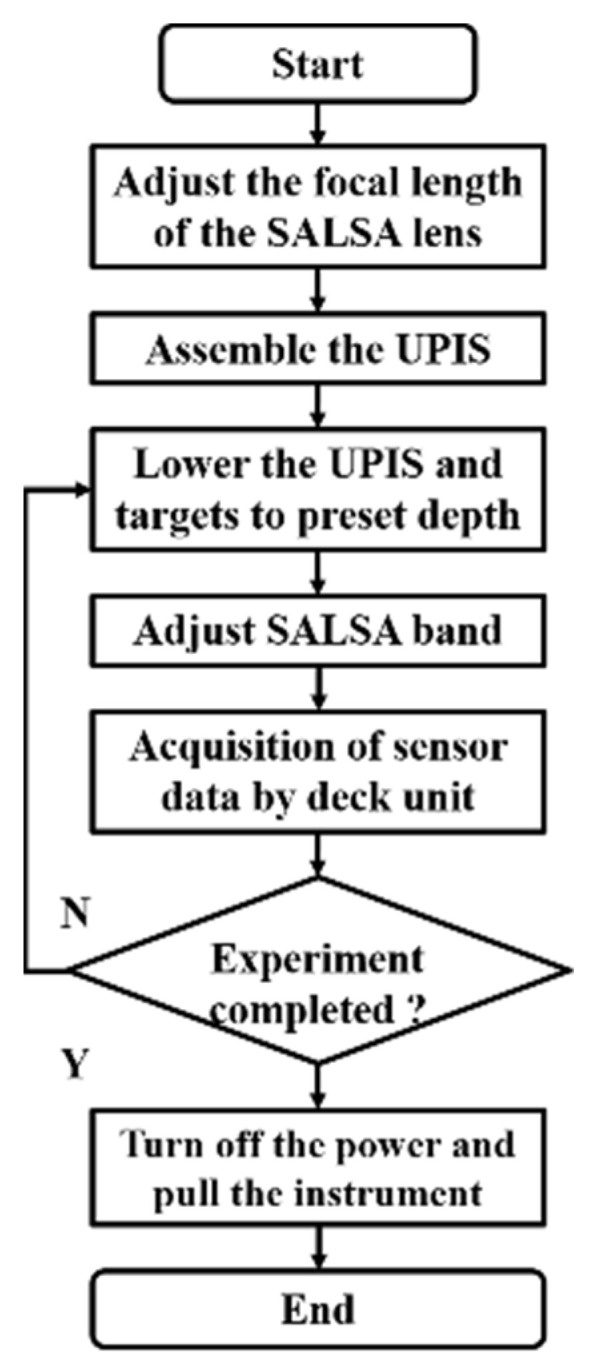
Flowchart of the experiment.

Referring to the flowchart (Figure 4) and water depth record (Table 2), we changed the depth of the target and the UPIS, and we conducted bottom-up polarization imaging of the target in Qiandao Lake to obtain images of *S*_0_, *S*_1_, *S*_2_, *S*_3_, DOP, DOLP, DOCP, AOP, and *ε*;.

Water absorption and scattering are wavelength dependent, especially absorption, which leads to obvious differences in the imaging capability at different wavelengths in underwater polarization imaging. In the experiment, to compare the performance of different bands, eight SALSA sampling bands were tested (Table 1). By comprehensively comparing the imaging quality of polarization parameters, it was determined that the band most suitable for polarization detection was the blue–green band (550 nm). This was principally due to the weak absorption and scattering of blue–green light at the MT site of Qiandao Lake, bringing about strong light penetration. In other words, the polarization imaging quality of targets received by the SALSA camera at 550 nm is the best among the available bands.

**Figure 5:**
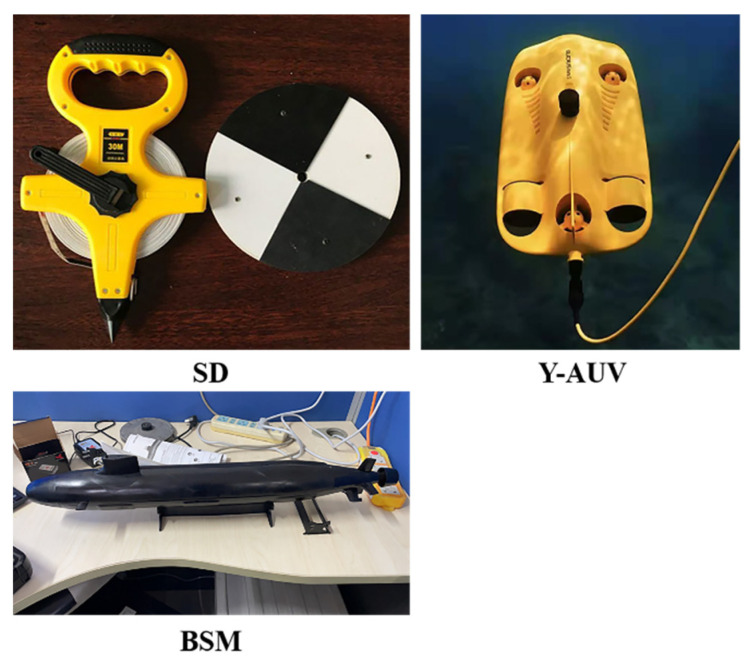
Targets used in Experiment 2.

In Experiment 2, underwater imaging of the UPIS was studied using three artificial targets: a Secchi disk (SD), a black ship model (BSM), and a yellow autonomous underwater vehicle (Y-AUV) (see Figure 5). It should be noted that, during the experiment, to clearly image the SD using the UPIS, the black-and-white side was downward, and the non-black-and-white side was upward, so that the SD could be clearly and intuitively identified in the real-time imaging when targets were observed from the bottom up. The reasons for using these objects were as follows: The SD was arranged to reflect the ability of UPIS to distinguish the target with a simple black-and-white segmentation line from bottom up. Compared with the SD, there was not clearly segmentation line on the surface of the Y-AUV, while performed the capacity of Y-AUV to bottom-up polarization image targets with complex contour features. Then, the BSM surface was coated with highly absorption material such as black matte paint, and there were no complex contour features or evident segmentation on the body. In the background of water surface while UPIS imaging from bottom up, identifying the BSM was the most arduous among the three artificial targets.

## 4. Results

### 4.1. Same Target with Different Depths

In this experiment, the relative depth of the UPIS and the SD remains unchanged. The relative depth was set to 1.0 m, as determined by the focal length of SALSA lens. By synchronously changing the depth of the UPIS and the SD in the water at the MT site, the capacity of the UPIS to detect the target from bottom up under different light field intensities was examined.

**Figure 6:**
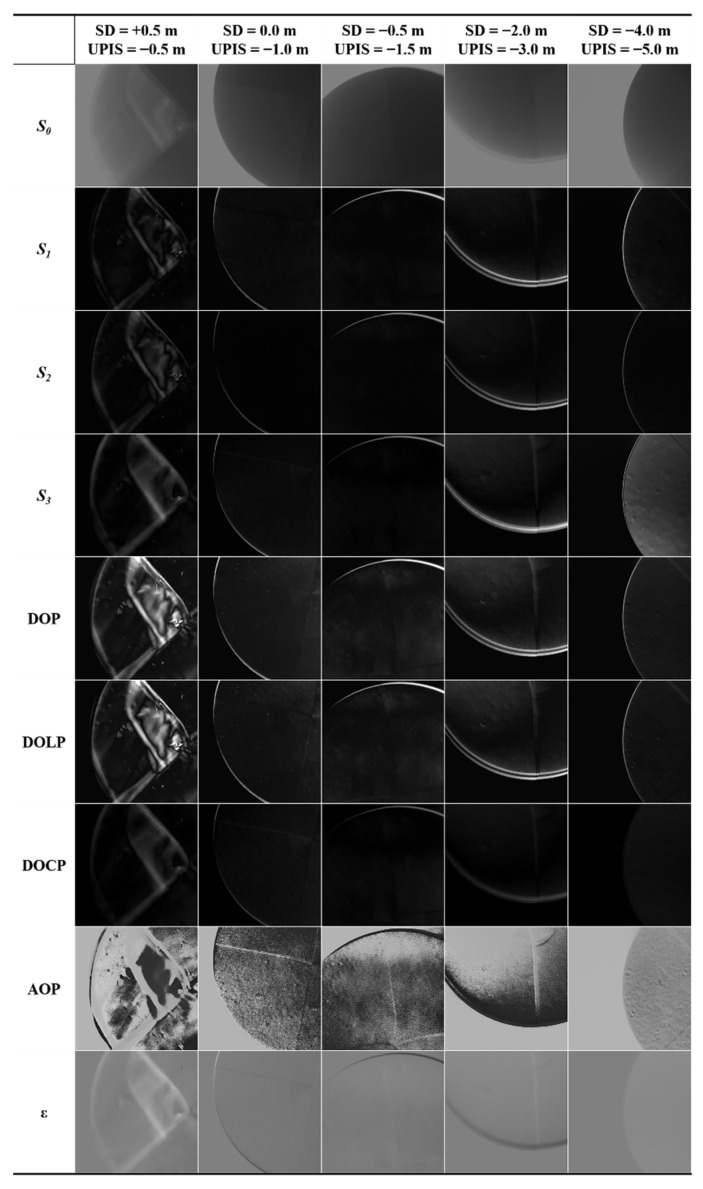
Variation of imaging parameters with the depth of the SD and the UPIS.

Figure 6 shows the change of each imaging parameter that occurred by changing the depth of the SD and the UPIS. With the deepening of the SD and the UPIS in the water, the intensity of the surrounding light field gradually weakened, resulting in a gradual increase in the difficulty of underwater target detection. At the beginning, the SD was 0.5 m above the water surface, and the UPIS was 0.5 m below the water surface. It can be seen from the *S*_0_ image that the light field condition was proper at this time. The total light intensity parameter *S*_0_ and polarization parameters *S*_1_, *S*_2_, *S*_3_, DOP, DOLP, DOCP, AOP, and *ε*; clearly exhibit the edge information of the SD. With the deepening of depth, the light intensity around the SD and the UPIS gradually weakened, and the intensity of the upward radiation in water entering the SALSA lens after being reflected by the surface below the SD further weakened. With increasing depth, it became more and more difficult to identify the black-and-white segmentation line of the SD by just using *S*_0_. When the SD was 4.0 m underwater and the UPIS was 5.0 m underwater, it became almost impossible to make out the black-and-white segmentation line of the SD simply through intensity only. Relatively, the performance of polarization parameters for target detection gradually improved with increasing depth, especially the AOP, which can clearly distinguish the black-and-white segmentation line of the SD. Compared with the *S*_0_ image, the edge obtained using polarization parameter imaging is clearer; that is, the target recognition capability is stronger.

**Figure 7:**
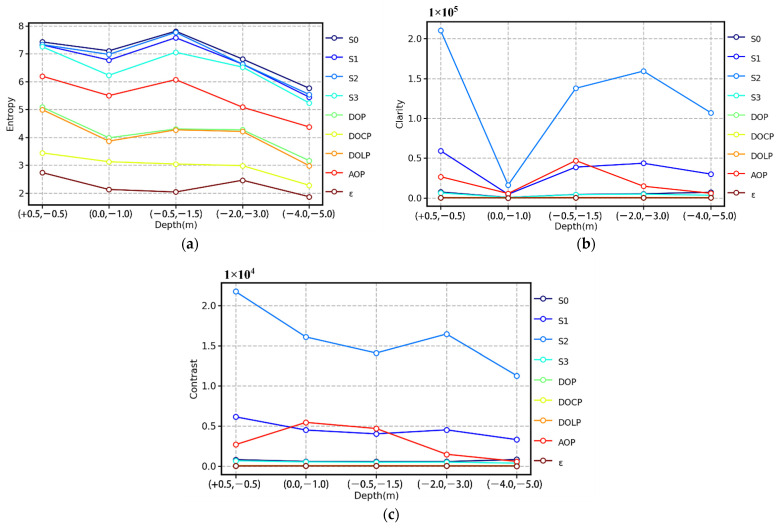
(**a**) Entropy, (**b**) clarity, and (**c**) contrast variation with the depth of the SD and the UPIS. The *x* coordinate indicates the depth of the SD or the UPIS. The *y* coordinate indicates the evaluation index value of image quality.

On the basis of Figure 6, we can easily work out that the capability of the AOP in terms of underwater target recognition is prominent at different water depths. Analytically, according to Equations (9)–(11), the entropy, clarity, and contrast all the parameters can be calculated, respectively, as shown in Figure 7. Because *S*_0_ is strongly affected by the absorption and scattering of particles in water, the *S*_0_ image will contain considerable background noise, which is evident in Figure 7a. Figure 7a shows that the entropy of *S*_0_, *S*_1_, *S*_2_, *S*_3_, and AOP is higher, whereas that of other parameters is relatively lower. In Figure 7b,c, the parameters with high clarity and contrast are also *S*_0_, *S*_1_, *S*_2_, *S*_3_, and AOP, but the performance of *S*_0_ is apparently worse, while the performance of AOP, *S*_1_, and *S*_2_ is still excellent. These results demonstrate that polarization imaging can indeed improve the ability of underwater target detection. Moreover, in the case of a weak light field, combined with Figure 6, the AOP is more noticeable than *S*_1_ and *S*_2_ visually.

### 4.2. Different Targets with the Same Depth

**Figure 8:**
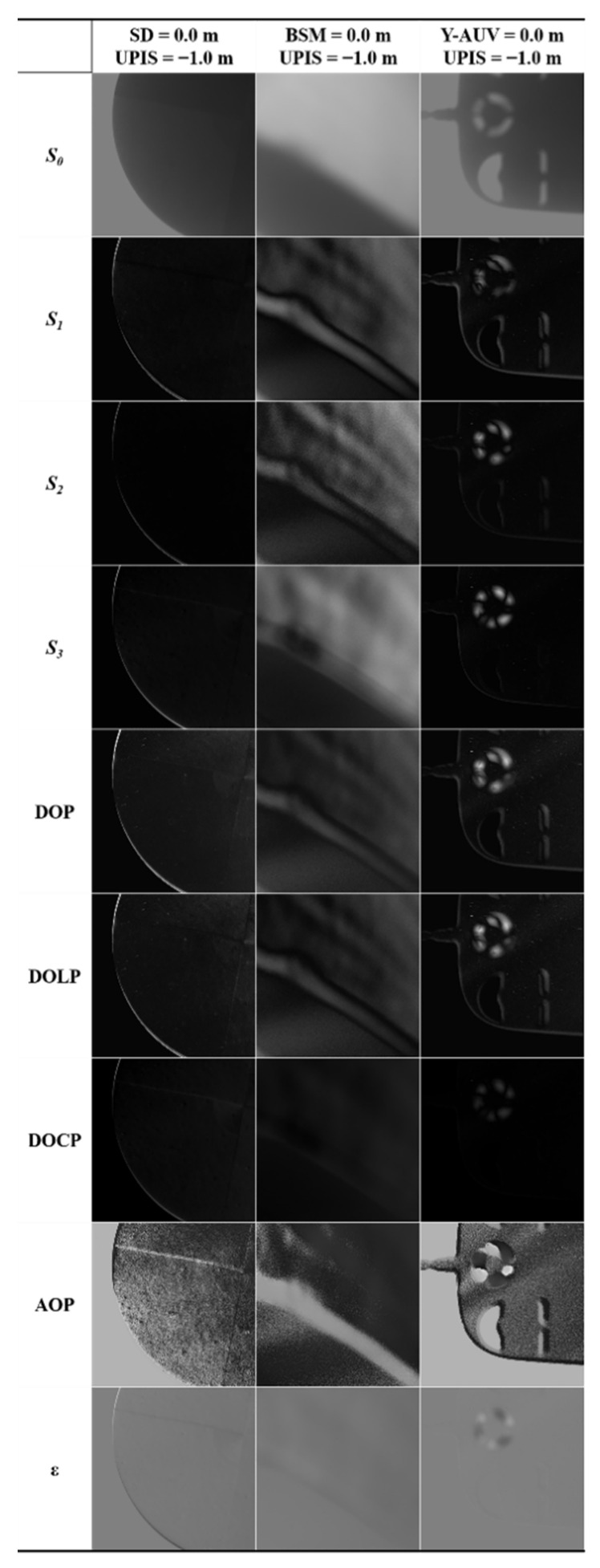
Variation of imaging parameters with the three kinds of targets.

**Figure 9:**
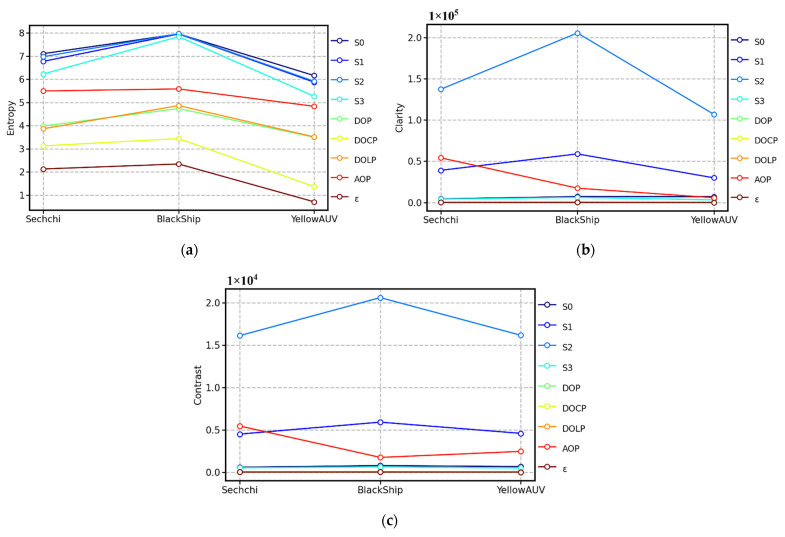
(**a**) Entropy, (**b**) clarity, and (**c**) contrast variation with three kinds of targets. The *x* coordinate indicates the different targets (SD, BSM, and Y-AUV). The *y* coordinate indicates the evaluation index of image quality.

Figure 8 shows *S*_0_, *S*_1_, *S*_2_, *S*_3_, DOP, DOLP, DOCP, AOP, and *ε*; corresponding to the SD, BSM, and Y-AUV when the targets were located on the water surface and the UPIS was located 1 m below the water surface. Generally, other cameras acquire images only through the intensity (*S*_0_, the first component of the Stokes vector). In contrast, the UPIS could imagine targets not only by intensity, but also by different polarization parameters. As could be seen from Figure 8, AOP imaging clearly distinguished the black and white lines of SD in field real-time imaging, while other imaging parameters have unsatisfactory ability to distinguish the black and white lines. It can be seen that images of AOP contain abundant details, where the texture and edge of targets under water are clearly highlighted. Compared with the SD, there was not clearly segmentation line on the surface of the Y-AUV, while performed the capacity of Y-AUV to bottom-up polarization image targets with complex contour features. For Y-AUV, the field imaging quality varied greatly with different parameters. AOP could not only distinguish the interface between Y-AUV and water, but also distinguish the color of Y-AUV body from the surrounding. Other parameters were not only inferior to AOP in the boundary differentiation of field real-time imaging, but also the color of Y-AUV body was almost the same as that of surrounding waters. Although the *S*_0_ could also distinguish the body and boundaries of Y-AUV, it was not as sharp and contrasting as AOP, as shown in Figure 9. In Figure 8, it was evident that *S*_0_ could not recognize waves caused by the agitation of the traction line, while DOCP and *ε*; could hardly distinguish the boundary between the BSM and the surrounding environment. In Figure 9, the entropy, clarity, and contrast of the imaging showed that AOP performed excellent among all parameters when identifying BSM. This further indicates that, for underwater target imaging observed from bottom up, the imaging quality of the AOP is optimal for these types of targets.

Similarly, to objectively evaluate the polarization image quality of these parameters, entropy, clarity, and contrast were calculated according to Equations (9)–(11). As shown in Figure 9, among the three objects, the entropy of the BSM was the highest and the texture and edge of the BSM were somewhat clearer in terms of clarity and contrast. We suspect that this is mainly because of the multiple wavy black lines in the BSM image in Figure 8. The wavy black lines appearing in the polarization images of Figure 8 are most likely caused by the agitation of the traction rope. In the in situ experiment, the light-weight BSM cannot move autonomously, and it mainly moves laterally through the traction rope. Relatively, the SD relies on the scale rope for vertical motion, and the Y-AUV can be remotely controlled for both horizontal and vertical motions. Therefore, once there is a strong flow fluctuation around the BSM, such as the agitation caused by the traction rope, obvious wavy black lines will appear in the polarization image, which will lead to high values of entropy, clarity, and contrast. This further demonstrates that polarization detection has high sensitivity and can capture the changes around the target in detail.

## 5. Discussion

In this study, the UPIS was used to detect the target from bottom up in Qiandao Lake. The experimental results show that polarization parameter imaging has a superior capacity to identify textures and edge of targets than intensity-only imaging and that the AOP exhibits excellent performance in these experiments. Compared with intensity-only imaging, underwater target polarization imaging not only contains sufficient information but also offers higher clarity and contrast (see Figure 7 and Figure 9).

**Figure 10:**
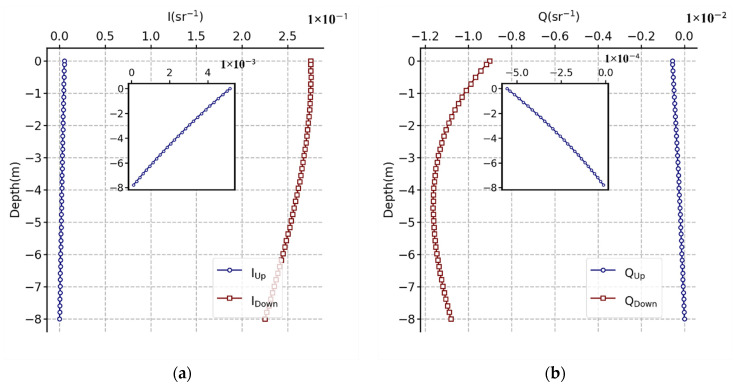

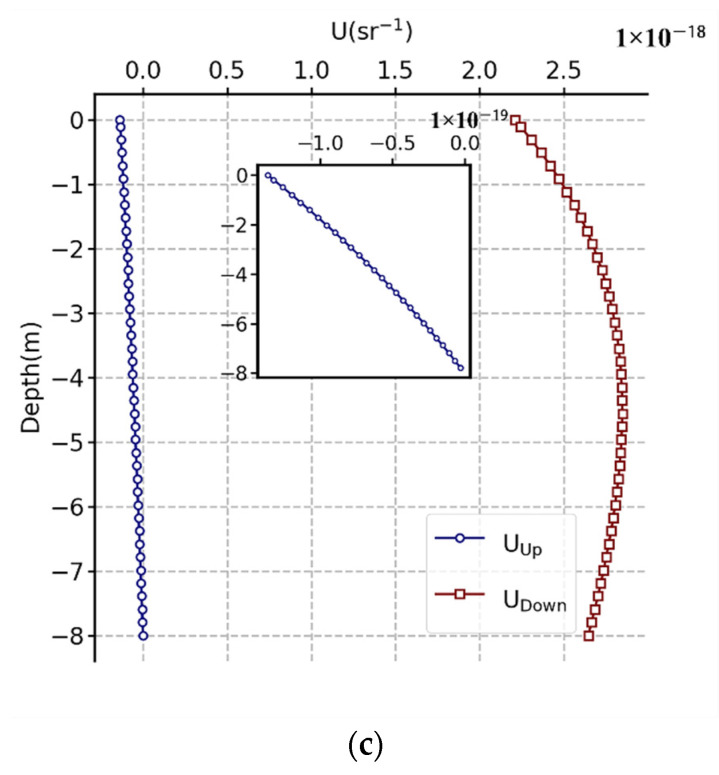
(**a**) *I*, (**b**) *Q*, and (**c**) *U* variation with water depth based on the OSOAA radiative transfer model simulation. The in situ water depth was 8 m. The solar zenith angle and viewing zenith angles were 45° and 0°, respectively. The simulated wavelength was 550 nm. The chlorophyll concentration at the water surface was 1.02 $\mathrm{mg}\cdot m^{-3}$ without regard to mineral-like particles and yellow substances and detritus. The aerosol optical thickness was 0.611, and M90 of the Shettle and Fenn model [30] was adopted.

**Figure 11:**
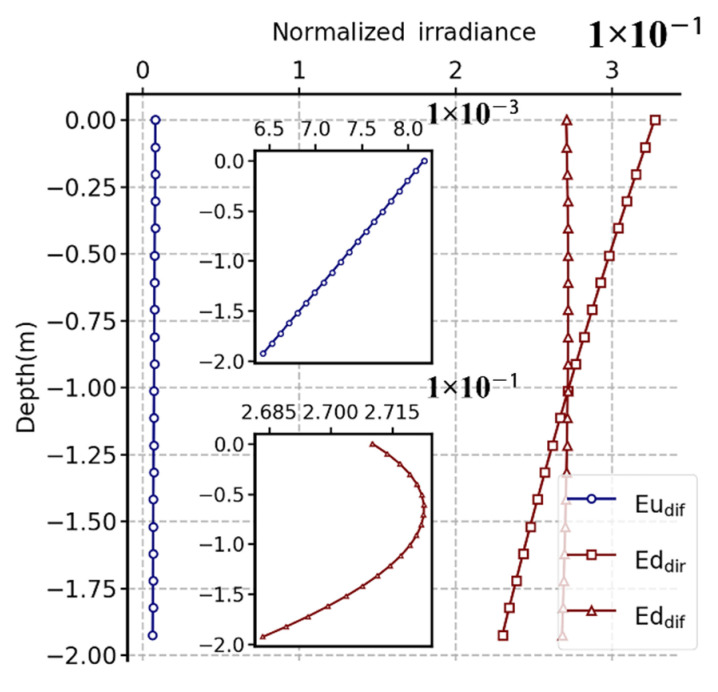
Underwater upward diffuse irradiance (Eu_dif_), downward direct irradiance (Ed_dir_), and downward diffuse irradiance (Ed_dif_) variation with water depth based on the OSOAA radiative transfer model simulation. Only the depth range with the target and the UPIS is shown.

When light propagates in water, the intensity of light decreases rapidly with the increase in depth because of absorption and scattering. Through subjective visual and objective image evaluation indexes (Figure 6 and Figure 7), it can be concluded that, with depth increasing, the information contained in the image exhibited a decreasing trend as a whole, and the clarity and contrast of the image also decrease. This is mainly because the total light intensity decreases with increasing depth, and the portion of light entering the polarization sensor lens through scattering with water will also decrease. In Figure 10, we used the OSOAA radiation transmission model to simulate the distribution of the underwater light field based on the condition of field parameters. OSOAA calculates the ocean–atmosphere coupled vector radiative transfer equation by using the successive scattering method [29]. It can be seen that, with the increase in depth, whether for *S*_0_ or the polarization components *S*_1_ and *S*_2_ (and *S_3_* ≈ 0), in terms of absolute value, the upward part is far less than the downward part, which means that the radiation entering the polarization camera lens after the upward light reflected by targets is far less than the radiation of its background light field (namely, the downward radiation), which is valid for both *S*_0_ and *S*_1_ and *S*_2_. Meanwhile, to verify this point, Figure 11 shows the corresponding upward and downward irradiance changes with depth. The figure shows that upward diffuse irradiance is far less than downward diffuse irradiance and direct irradiance. Furthermore, this is also one of the key difficulties for target detection from bottom up in water relative to target detection from top down. When sensors detect targets underwater, the intensity of light containing the shape information of targets received by sensors from bottom up was much lower than that under the same conditions received by sensors from top down. Therefore, when using the total light intensity parameter *S*_0_ for detection, the segmentation line between the target and the background can be easily identified, but the texture features of the target cannot be clearly identified. Currently, this problem can be solved for polarization images, especially for polarization images calculated by using *S*_1_ and *S*_2_. When the light is reflected on the surface of different targets, the polarization state of the reflected light will change. As a consequence, while the part representing intensity is eliminated in the process of calculating the polarization parameters, the part of the polarization state caused by the change of the shape and texture of the target will be prominent, which is the reason why some polarization parameters can more clearly image the texture characteristics of underwater targets relative to *S*_0_.

Generally, because the underlying surface of an underwater target usually reflects light diffusely and the direct light is occluded by the target, the incident light is approximately isotropic after diffuse reflection on the target surface. That is, the reflection light *S*_0_ exists, but *S*_1_ and *S*_2_ are negligible. However, in the UPIS target detection experiments, the reason why the texture and edge of underwater targets can be identified so well by polarization parameters, especially the AOP, is that the shape information of targets is still mainly contained in *S*_1_ and *S*_2_, while *S*_1_ and *S*_2_ are non-negligible after the reflection. In other words, the polarization diffuse reflectance is not the same at different positions on the target surface, and *S*_1_ and *S*_2_ of the light obtained by the upward light after diffuse reflection on the target surface do not completely disappear, which can be inferred from Figure 6 and Figure 8.

We consider ignoring inelastic scattering, the chlorophyll fluorescence effect under water, and the direct light that is occluded by the target. Then, we can obtain the following equation of the underwater radiation transmission:

(12) $$\vec{L}\left(z\right)=\vec{M}\cdot \vec{L}\left(0^{-}\right),$$

where $\vec{L}\left(z\right)$ denotes ${\left[S_{0},\;S_{1},S_{2}\right]}_{z}^{T}$ at the underwater depth of *z* meters, $\vec{L}\left(0^{-}\right)$ denotes ${\left[S_{0},\;S_{1},S_{2}\right]}_{z=0^{-}}^{T}$ at the subsurface of the water, and $\vec{M}$ denotes the Muller matrix connecting scattering and incident radiation. By considering that water particles are uniform and spherical and that the angles of incident light and scattered light are extremely close to 0° (i.e., only the dominant forward scattering part is considered), we can obtain the following simplified expression for the Muller matrix:

(13) $$\vec{M}=\left[\begin{array}{ccc} m_{11} & m_{12} & 0\\ m_{12} & m_{11} & 0\\ 0 & 0 & m_{33} \end{array}\right].$$

According to the definition of the Muller matrix, each element mainly represents the polarization tendency of scattered light when the incident light is polarized [31]. Meanwhile, when the underwater characteristic parameters of water are constant and the observation azimuth is constant, the Muller matrix will not change with the incident light. Combining this result with Equation (12), we can obtain

(14) $$\{\begin{array}{l} I_{z}=m_{11}\cdot I_{0}+m_{12}\cdot Q_{0}\\ Q_{z}=m_{12}\cdot I_{0}+m_{11}\cdot Q_{0}\\ U_{z}=m_{33}\cdot U_{0} \end{array}.$$

Solving this set of equations for *m*_11_ and *m*_12_ and *m*_33_ gives

(15) $$\{\begin{array}{l} m_{11}=\frac{I_{z}\cdot I_{0^{-}}-Q_{z}\cdot Q_{0^{-}}}{I_{0^{-}}^{2}-Q_{0^{-}}^{2}}\\ m_{12}=\frac{Q_{z}\cdot I_{0^{-}}-I_{z}\cdot Q_{0^{-}}}{I_{0^{-}}^{2}-Q_{0^{-}}^{2}}\\ m_{33}=\frac{U_{z}}{U_{0^{-}}} \end{array}.$$

According to the downward-transmitted normalized Stokes vector simulated by OSOAA (Figure 10), we can obtain

(16) $$\{\begin{array}{l} I_{z=0^{-}}=0.275324\\ I_{z=1}=0.275223\\ Q_{z=0^{-}}=-0.0090174\\ Q_{z=1}=-0.0101897\\ U_{z=0^{-}}=2.20855\times 10^{-18}\\ U_{z=1}=2.49569\times 10^{-18} \end{array}.$$

Then, combined with Equation (15), the Muller matrix from the subsurface to 1 m underwater can be solved as follows:

(17) $$\vec{M}=\left[\begin{array}{ccc} 0.999493 & -0.004274 & 0\\ -0.004274 & 0.999493 & 0\\ 0 & 0 & 1.130013 \end{array}\right].$$

After calculating the Muller matrix, we can obtain the value of ${\left[S_{0},\;S_{1},S_{2}\right]}_{z=1}^{T}$ measured by the UPIS at 1 m underwater after the diffuse reflection of the target. If the diffuse reflectance distribution on the target surface is ${\vec{R}}_{dif}\left(\vec{x}\right)$, the radiation received by the UPIS at 1 m underwater can be expressed as follows:

(18) $$L\left(z\right)=\vec{M}\cdot {\vec{R}}_{dif}\left(\vec{x}\right)\cdot L\left(0^{-}\right).$$

Then, we combine Equation (18) with Equations (16) and (17) and finally obtain the following expressions for *S*_0,z = 1m_, *S*_1,z = 1m_, and *S*_2,z = 1m_:

(19) $$\{\begin{array}{l} S_{0,\;z=1m}=p_{11}\cdot S_{0,\;0^{-}}\cdot r_{I}+p_{12}\cdot S_{1,\;0^{-}}\cdot r_{Q}=0.275284r_{I}+3.854481\times 10^{-5}r_{Q},\\ S_{1,z=1m}=p_{12}\cdot S_{0,\;0^{-}}\cdot r_{I}+p_{11}\cdot S_{1,\;0^{-}}\cdot r_{Q}=-0.001177r_{I}-0.009013r_{Q},\\ S_{2,z=1m}=p_{33}\cdot S_{2,\;0^{-}}\cdot r_{U}=2.495690\times 10^{-18}r_{U}. \end{array}.$$

As a result of the depolarization of diffuse reflection, $r_{I}\gg r_{Q}\;\mathrm{and}\;r_{I}\gg r_{U}$; that is,

(20) $$\frac{r_{Q}}{r_{I}}\to 0\;\mathrm{and}\;\frac{r_{U}}{r_{I}}\to 0.$$

By analyzing the above equations and substituting them into the calculation formulas of each polarization parameter from Equations (4)–(8), we can obtain

(21) $$\mathrm{DOP}=\frac{\sqrt{S_{1}^{2}+S_{2}^{2}+S_{3}^{2}}}{S_{0}}\times 100\%\approx \frac{\sqrt{{\mathrm{Const}}_{1}^{2}\cdot r_{I}^{2}}}{r_{I}}\times 100\%={\mathrm{Const}}_{1}\times 100\%,$$

(22) $$\mathrm{DOLP}=\frac{\sqrt{S_{1}^{2}+S_{2}^{2}}}{S_{0}}\times 100\%\approx \frac{\sqrt{{\mathrm{Const}}_{2}^{2}\cdot r_{I}^{2}}}{r_{I}}\times 100\%={\mathrm{Const}}_{2}\times 100\%,$$

(23) $$\mathrm{DOCP}=\left|\frac{S_{3}}{S_{0}}\right|=0,$$

(24) $$\mathrm{AOP}=\frac{1}{2}\arctan\frac{S_{2}}{S_{1}}=\frac{1}{2}\arctan\left({\mathrm{Const}}_{4}\cdot \frac{r_{U}}{r_{Q}\;}\right)\;\left(\mathrm{in}\;\mathrm{degrees}\right),$$

(25) $$\varepsilon =\frac{1}{2}\arcsin\frac{S_{3}}{S_{1}}=0.$$

Comparing the expressions of various polarization parameters above, we find that only the AOP can effectively reflect the polarization diffuse reflectance information of different positions on the surface of the target and that the values of other polarization parameters are very close to a constant because $r_{I}\gg r_{Q}\;\mathrm{and}\;r_{I}\gg r_{U}$ on the target surface. Moreover, this also explains why (see Figure 6, Figure 7, Figure 8 and Figure 9) the AOP offers the best performance among polarization parameters. Therefore, because of the different polarization diffuse reflectance values of the target surface (i.e., the varying distribution of $r_{U}/r_{Q}$ on the target surface), the texture and edge of the target can be correctly identified by using the AOP.

In terms of multi-parameter comprehensive utilization in practical application, we would recommend using AOP in combination with *S*_0_ for bottom-up detection of underwater targets. There are two reasons for this choice. First, as the result of the depolarization of diffuse reflection on the target surface, DOP, DOLP, DOCP and *ε* keep close to constant (see Equations (20)–(25)), which makes them almost impossible to reflect the texture characteristics of the target surface. Meanwhile, AOP is computed by *S*_1_ and *S*_2_, and contains the major information about target texture of linear polarization component. Therefore, considering the validity and incoherence of imaging parameters, *S*_0_ is a recommended option for detection combined with AOP if necessary. Second, from the perspective of entropy (see Figure 7a and Figure 9a), *S*_0_ is the prominent imaging parameter containing the most information among all the used parameters. Combined with the outstanding performance of AOP in clarity and contrast (see Figure 7b,c and Figure 9b,c), it may be possible to extract more effective new texture information of targets from *S*_0_. It is natural to recognize that polarization imaging for SS (semantic segmentation) will be a valuable and pioneering work. Due to the different diffuse reflection parameters of the surface of different materials, the polarization states of the light reflected from the surface of different targets will have obvious differences and correspond with the materials. Xiang et al. [32] built the first RGB-P dataset, which consists of 394 annotated pixel-aligned RGB-polarization images and showed the effectiveness of EAFNet (the Efficient Attention-bridged Fusion Network) to fuse polarization and RGB information to examine SS through various experiments. For future work, we will further carry out the SS for underwater targets from bottom up.

## 6. Conclusions

In this study, we examined the imaging detection capacity of a polarization camera for observing a target underwater from bottom up. First, based on the SALSA polarization camera, we developed a UPIS to realize bottom-up detection of underwater targets based on polarization imaging. UPIS could carry out multi-band polarization imaging detection of underwater targets within 50 m of water depth. Then, using the UPIS, we conducted imaging bottom-up detection experiments to observe the target in water in the relatively clear Qiandao Lake, and we recorded polarization data at different depths and for different targets. The field results showed that *S*_0_ could detect targets with shallow water depth under good light field conditions, but it was not suitable for the field of weak environmental light. The imaging capacities of polarization parameters were relatively stable in the process of deepening water depth. Among them, AOP had prominent polarization imaging ability in both experiments of deepening water depth and different types of targets. In addition, through objectively comparing the entropy, clarity, and contrast of different polarization parameters, the AOP was determined to offer the best performance for imaging targets from bottom up.

Based on the underwater light field distribution at different depths obtained by the OSOAA simulation, we theoretically derived the formulas for each polarization parameter when detecting from bottom up. The theoretical results demonstrate that, because of the different polarization diffuse reflectance values of the target surface (i.e., the varying distribution of $r_{U}/r_{Q}$ on the target surface) and the relationships $r_{I}\gg r_{Q}\;\mathrm{and}\;r_{I}\gg r_{U}$ on the target surface, the AOP is the most suitable for target polarization imaging detection from bottom up among the parameters investigated in this study. Unlike the numerical simulation study, our in situ experiment was only carried out at the Qiandao Lake, which is a clear water lake. In future work, more experiments under different conditions should be carried out to comprehensively test the polarization imaging ability of underwater targets.

## Figures and Tables

**Table 1:** SALSA polarization camera parameters.

Parameter	Value
Camera size (mm × mm × mm)	80 × 80 × 100
Resolution (pixel × pixel)	782 × 582 to 1040 × 1040
Frame rate (frame∙s^−1^)	12 (12 bits); 20 (8 bits)
Bit number for each pixel	8 or 12
Access port	USB
Central wavelength for each band (nm)	410, 443, 490, 520, 550, 620, 660, 685
Lens focal length (mm)	77
Software	SALSA version 2.3.6

**Table 2:** The depths of target and UPIS in Experiment 1, with “+” and “−” represent above and below water surface, respectively.

Target Depth (m)	UPIS Depth (m)
+0.5	−0.5
0.0	−1.0
−0.5	−1.5
−2.0	−3.0
−4.0	−5.0

## Data Availability

All data generated or that appeared in this study are available upon request by contact with the corresponding author. Furthermore, the models and code used during the study cannot be shared at this time as the data also form part of an ongoing study.
